# Syphilis in Its Medical, Medico-legal and Sociological Aspects

**Published:** 1908-07

**Authors:** 


### BOOKS AND AUTHORS.

---

**Syphilis in its Medical, Medico-legal and Sociological Aspects.** By **A. Ravogli,** M.D., Professor of Dermatology and Syphilology in the Medical College of Ohio, Medical Department of Cincinnati University. Octavo, pp. 518. Illustrated. New York: The Grafton Press.

The value of this book cannot be overestimated, and it will rank as an authority on the medicolegal aspect of syphilis. It is most complete in construction and there is apparently nothing lacking to place it among the foremost works in syphilographic literature. The fore portion of the book is devoted to syphilis as a disease; the search for the cause of the affection, the processes and modes of infection; the evolution of the disease and its attack on the various parts of the body; and, moreover, there is considerable space devoted to the various methods of treatment of the different manifestations of the disease. Whatever one may personally believe in as regards treatment, he must admit that the general plan as laid out by Ravogli is conservative and sane and that in the majority of uncomplicated cases it will bring about a cure of the condition.

The chief value of the book, however, lies in the section devoted to the medicolegal and sociologic aspects of syphilis, and because of the inestimable value of this portion of the work it is entitled to the highest consideration and should be given at least a careful reading by every man who meets with the disease in practice. The question of marriage and syphilis is gone into at great length, and the relationship of the combination as a menace to public health is clearly pointed out. Furthermore, every legal authority bearing on the subject of venereal disease, in which is included gonorrhea and syphilis, is quoted in its direct application to divorce.

The author points out with cameo distinctness the visible evidences of degeneracy as a result of syphilis and sums up the work with a series of recommendations looking to the control of the disease under consideration, insofar as society may be controlled in the exhibition of its inclinations, its habits, or its individual egotistic prowess. Of course, there are any number of hypersensitive purists who will raise their eyes to heaven making vociferous objection to the proposition, that every man who applies for a marriage license shall undergo a physical examination to determine the presence or absence of any of the venereal diseases, before the license shall be granted. Yet, all things considered, this is the most certain method of limiting the scope of gynecologic activity; always, however, assuming that the post marital life of the individual shall be one of spotless sexual sanctity.

At the same time, a more or less extensive experience in the field of genitourinary work leads the writer to suggest as an amendment, that the gentle member of the proposed life partnership be also included in this physical examination, for it would appear that there is quite as much infection in female vaginas as there is in male urethras. The average purist is too delicately nice to take serious cognisance of venereal disease except in its application to prostitution, and in his analysis of the mud in the streets he pays little attention to the slush which comes from the gutters above his head; this is an error which Ravogli does not fall into. Regulation of prostitution which is considered rather fully in the book, is theoretically an excellent thing—for the police department of any large city; but the regulation of the necessary evil in European cities has not been productive of any very great degree of sexual cleanliness. On the other hand, there has not been a church movement for reform in this country within recent years, where the reform was directed specifically against prostitutes, which has not been followed by an outbreak of venereal disease and increased the drug store sales of "sure cures" to a most satisfying extent. Dr. Ravogli has given a most excellent work to medical literature and his book may be read with advantage, not only by physicians, but by lawyers, and incidentally by the reform-bitten preacher.

